# Novel insights from a large cohort: Elucidating incidence, risk factors, treatment, and prognostic predictors in autoimmune hemolytic anemia after allogeneic hematopoietic stem cell transplantation

**DOI:** 10.1515/jtim-2026-0030

**Published:** 2026-04-04

**Authors:** Zhuoyu An, Liqian Zhang, Peng Zhao, Jin Wu, Haixia Fu, Yuanyuan Zhang, Xiaodong Mo, Fengrong Wang, Chenhua Yan, Yuqian Sun, Meng Lv, Yuhong Chen, Yingjun Chang, Yu Wang, Lanping Xu, Xiangyu Zhao, Xiaojun Huang, Xiaohui Zhang

**Affiliations:** Peking University People's Hospital, Peking University Institute of Hematology, Beijing, China; National Clinical Research Center for Hematologic Disease, Beijing, China; Beijing Key Laboratory of Cell and Gene Therapy for Hematologic Malignancies, Peking University, Beijing, China

**Keywords:** autoimmune hemolytic anemia, allogeneic hematopoietic stem cell transplantation, risk factors, treatment, prognostic predictors

## Abstract

**Background and Objectives:**

Autoimmune hemolytic anemia (AIHA) is a rare but serious complication after allogeneic hematopoietic stem cell transplantation (allo-HSCT). Current understanding of post-allo-HSCT AIHA remains insufficient. This study aimed to elucidate the features and significant predictors of post-allo-HSCT AIHA to guide precise clinical management.

**Methods:**

A retrospective nested case-control study was conducted at Peking University People's Hospital from 2013 to 2024. A total of 61 patients with post-allo-HSCT AIHA were enrolled. For each case, three AIHA-free allo-HSCT recipients were randomly matched by sex, age (± 3 years), and transplant timing (± 3 months). Treatment modalities and responses of AIHA patients were analyzed. Cox regression analyses were employed to identify risk factors for post-allo-HSCT AIHA, as well as predictors of AIHA relapse and patient mortality.

**Results:**

The incidence of post-allo-HSCT AIHA was 0.72%. During follow-up, 14 (22.95%) AIHA patients died (mostly because of severe infection-related complications) and 6 (9.84%) experienced AIHA relapse. Multivariate analysis showed that an unrelated donor was an independent risk factor for post-allo-HSCT AIHA (Hazard Ratio [HR] 2.323, *P* = 0.027), whereas a history of acute graft-versus-host disease was a protective factor (HR 0.340, *P* = 0.001). Corticosteroids combined with rituximab significantly increased the likelihood of treatment response (*P* = 0.003), with no significant adverse reactions or treatment-related mortality observed. We revealed that mononuclear cell count at allo-HSCT was correlated with AIHA relapse (HR 1.720, *P* = 0.011). Age (per 10-year increase, HR = 1.669, *P* = 0.005), lactate dehydrogenase (LDH) level (per 100-unit increase, HR = 1.178, *P* = 0.020), and C-reactive protein level (CRP, mg/L, HR = 1.018, *P* = 0.031) were associated with an increased risk of mortality.

**Conclusions:**

This study provides novel insights into post-allo-HSCT AIHA. These findings highlight key prognostic markers, guiding risk stratification and the management of AIHA after allo-HSCT.

## Introduction

Autoimmune hemolytic anemia (AIHA)is a rare yet serious hematologic disorder characterized by autoantibody-mediated destruction of red blood cells (RBCs), in which autoantibodies target antigens on the RBC surface.^[1,2]^ While allogeneic hematopoietic stem cell transplantation (allo-HSCT) is well recognized as the most effective therapeutic option for a wide range of malignant and nonmalignant hematological disorders, AIHA has been identified as an infrequent complication following allo-HSCT.^[3,4]^ The incidence of AIHA in patients who underwent HSCT varies among studies, ranging from less than 1% to 6%.^[5, 6, 7, 8, 9, 10, 11]^

On the basis of differences in clinical manifestations and antibody types, AIHA can be categorized into warm antibody-mediated AIHA (wAIHA), cold agglutinin disease (CAD), cold agglutinin syndrome (CAS), paroxysmal cold hemoglobinuria (PCH), and other rare subtypes.^[1,12]^ The incidence rates of different AIHA subtypes following allo-HSCT have not been fully clarified. Current evidence suggests that post-allo-HSCT AIHA is likely associated with post-transplant immune system dysregulation and immune intolerance.^[11,13,14]^ The risk factors for post-allo-HSCT AIHA reported in previous studies include underlying non-malignant diseases, blood group incompatibility (predominantly ABO incompatibility), the use of unrelated donors, human leukocyte antigen (HLA) mismatching, graft-versus-host disease (GVHD), and post-transplant infections.^[3,6,7,8,9,14,15]^ AIHA can significantly complicate post-transplant patient management and increase the risk of adverse prognostic outcomes.^[6,16]^ However, current therapeutic interventions for AIHA following allo-HSCT remain mainly derived from conventional AIHA treatments and lack robust clinical evidence for efficacy in this special context. In addition, few studies have identified prognostic predictors of AIHA following allo-HSCT. Therefore, studies identifying robust prognostic predictors of post-allo-HSCT AIHA are urgently needed to guide more precise clinical management and improve patient outcomes. Furthermore, key clinical and biological markers influencing relapse after remission or predicting ultimate mortality remain poorly understood, leading to a lack of effective risk-stratification strategies in clinical management.

In the present study, we used data from the largest cohort of AIHA patients after allo-HSCT to elucidate the associated clinical characteristics, risk factors, clinical outcomes, and prognostic predictors. This study systematically identified independent prognostic predictors for relapse and mortality in patients with post-allo-HSCT AIHA, which may facilitate more precise guidance for individualized risk-based management of these patients and thereby improve outcomes following allo-HSCT.

## Material and methods

### Study patients

**Figure 1:**
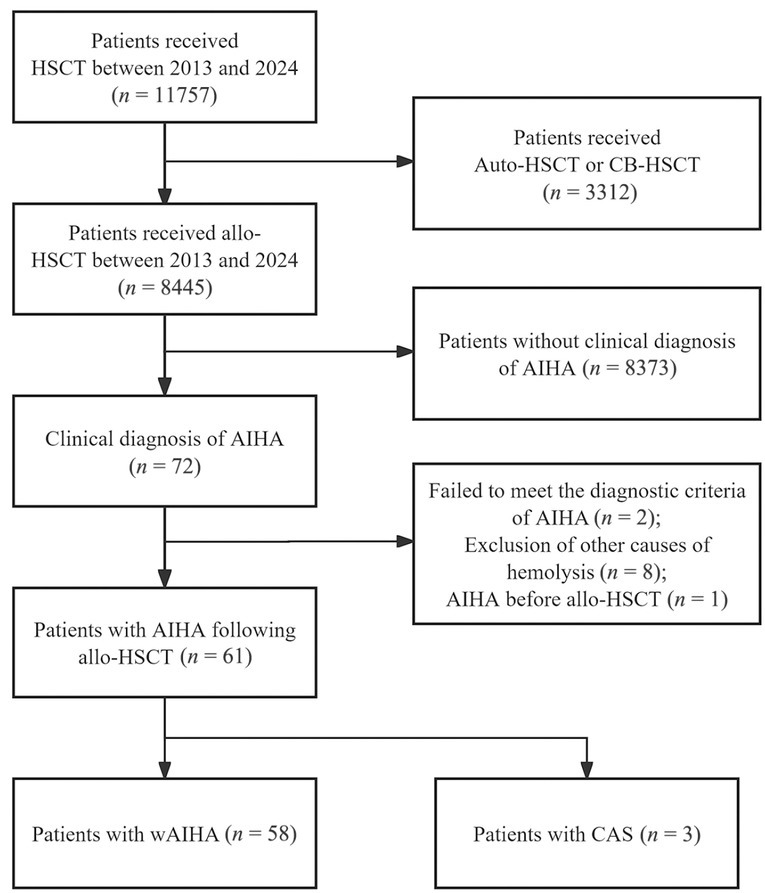
Flow chart of patient screening. HSCT: hematopoietic stem cell transplantation; Auto-HSCT: Autologous HSCT; CB-HSCT: cord blood HSCT; allo-HSCT: allogeneic HSCT; AIHA: autoimmune hemolytic anemia; wAIHA: warm antibody-mediated AIHA; CAS: cold agglutinin syndrome.

This was a retrospective, nested case-control study conducted from January 2013 to November 2024. A total of 8445 consecutive patients received allo-HSCT at Peking University People's Hospital. Based on medical records, 75 patients suspected of having AIHA were diagnosed with the condition. Patients who did not meet the diagnostic criteria for AIHA in this study and those who developed AIHA prior to allo-HSCT were excluded. Finally, 61 patients with AIHA following allo-HSCT were included in the case group (Figure 1). For each case, three AIHA-free allo-HSCT recipients were randomly selected from the 8445 patients to form the control group, which was matched on the basis of the following matching criteria: sex, age (± 3 years) , and timing of allo-HSCT (± 3 months) . Patients were followed for at least 24 months for relapse and overall survival (OS) *via* outpatient visits at the dedicated posttransplant clinic of our center. All enrolled patients submitted signed informed consent forms in compliance with the Declaration of Helsinki, and this study obtained approval from the Ethics Committee of Peking University People's Hospital.

### Conditioning therapy and prophylaxis of GVHD

In this study, the majority of patients received allografts obtained from partially HLA-matched donors and underwent transplantation using a previously described regimen.^[17, 18, 19, 20]^ This regimen comprised cytosine arabinoside (Ara-C, 4 g/m^2^/day on days −10 to −9), busulfan (BU, 3.2 mg/kg/day on days −8 to −6), cyclophosphamide (Cy, 1.8 g/m^2^/day on days −5 to −4), and anti-human thymocyte globulin (ATG, 2.5 mg/ kg/day on days −5 to −2); additionally, oral methyl-N-(2-chloroethyl)-N-cyclohexyl-N-nitrosourea (Me-CCNU) was given at a dose of 250 mg/m^2^ on day −3. Most HLA-matched recipients were administered the same regimen without ATG. Additionally, patients older than 55 years, with a hematopoietic cell transplantation-specific comorbidity index > 3, or who were intolerant to standard drugs, received reduced-intensity conditioning, which included the Bu/Cy/fludarabine (Flu)/ATG regimen and Bu/Cy/Flu regimen. For patients with specific clinical statuses (*e.g*., a history of prior transplantation), a conditioning protocol based on total body irradiation was adopted.^[18,21]^

For the prophylaxis of GVHD, a combination regimen was used, which included cyclosporine A (CsA), mycophenolate mofetil (MMF), and short-term methotrexate (MTX).^[22,23]^ CsA was intravenously administered at 2.5 mg/ kg per day starting on day −9; once the patient's bowel function returned to normal, the administration route was switched to oral administration. Serum CsA concentrations were monitored weekly, and dosage adjustments were made as required to maintain a minimum concentration of 150-250 ng/mL. A gradual reduction in CsA dose was then initiated in the absence of evidence of GVHD. MMF was administered orally at 0.5 g every 12 hours, starting from day −1 to day +30, while MTX was given intravenously at 15 mg/m^2^ on day +1 post-transplantation, followed by doses of 10 mg/m^2^ on days +3, +6, and +11. In instances where patients were intolerant to CsA, tacrolimus was used as an alternative, with a daily dosage of 0.02-0.03 mg/ kg and a treatment course lasting 3 to 6 months.^[19,22,23,24,25]^

### Definition

(1) AIHA was diagnosed based on the following criteria: anemia;^[26]^ evidence for hemolysis—including decreased serum haptoglobin, elevated total bilirubin (with a predominance of unconjugated bilirubin), increased serum lactate dehydrogenase (LDH), or either a reticulocyte percentage exceeding 4% or an absolute reticulocyte count above 120×109/L; a positive direct antiglobulin test (DAT); and ruling out of alternative causes (especially the hemolytic transfusion reaction identified by positive blood group antibodies).^[1]^

(2) The diagnostic criteria for wAIHA encompassed all the core AIHA diagnostic criteria, along with the absence of cold-related symptoms, a DAT positive for immunoglobulin G (IgG), immunoglobulin A (IgA), or complement 3d (C3d) ± IgG, and exclusion of clinically significant cold-reactive antibodies. CAD was diagnosed in AIHA patients who have a strongly positive DAT for C3d, a cold-agglutinin titre ≥ 64 at 4 ℃ and no identifiable underlying conditions as triggers, whereas CAS was defined by the same laboratory findings in the presence of an associated clinical condition. PCH was diagnosed in patients with autoantibody-mediated hemolysis and a positive Donath-Landsteiner test.^[1]^

(3) Treatment response included complete response (CR) and response (R). CR was defined as normalization of hemoglobin, no evidence of hemolysis (normal bilirubin, LDH, haptoglobin and reticulocytes), and freedom from transfusions. R was characterized by either a hemoglobin increase of > 2 g/dL or hemoglobin normalization (without complete biochemical resolution of hemolysis), with no transfusions administered in the preceding 7 days.^[1]^

(4) AIHA relapse was defined as the recurrence of disease manifestations meeting the diagnostic criteria for AIHA after achievement of CR or a sustained R lasting for more than four weeks. OS was defined as the time interval from the date of AIHA to death from any cause.

(5) Treatment-related adverse events reported in this study included infections and infestations (all episodes of bacterial, fungal, or viral infection that were either directly induced or clinically exacerbated by the treatment), blood and lymphatic system disorders (neutropenia or thrombocytopenia), gastrointestinal disorders (nausea, anorexia, vomiting, abdominal pain, diarrhea, or constipation), hepatobiliary disorders (elevated hepatic enzymes), nervous system disorders (headache, dizziness, hypoesthesia, paresthesia or tremor), renal disorders (elevated serum creatinine or other renal impairment evidence), and immune system disorders (allergic reactions or infusion-related reactions). The severity of each adverse event was graded according to the Common Terminology Criteria for Adverse Events version 5.0.

### Treatments of AIHA following allo-HSCT

For patients in whom AIHA was triggered by an underlying clinical condition, treatment of that condition was initiated promptly. In individuals with wAIHA or PCH, prednisolone or prednisone (1 mg/ kg/day) for 2-3 weeks was the first-line treatment and is applied immediately.^[1,2]^ If a sustained treatment response was achieved, the predniso(lo)ne was tapered over 4 weeks to 20.0-30.0 mg/ day and thereafter reduced by 2.5-10.0 mg/ day each month, with complete discontinuation within 3-6 months. For patients with severe anemia, inadequate response to or dependency on corticosteroids, corticosteroid dosages were further adjusted on the basis of the patient's clinical status and rituximab (RTX) was generally applied. The standard regimen for RTX was 375 mg/m^2^ once weekly for 4 consecutive weeks.^[27]^ Two alternative regimens were also available: a fixed high-dose regimen (RTX 1 g/ day, administered twice at a two-week interval) and a low-dose regimen (RTX 100 mg/ day, once weekly for 4 consecutive weeks).^[28, 29, 30]^ For patients with CAD or CAS, RTX was the preferred intervention, with the same regimen of 375 mg/m^2^ once weekly for 4 consecutive weeks. For AIHA patients with hemoglobin < 60 g/ L, the administration of intravenous immunoglobulin (IVIG, 0.4–0.5 g/ kg/day for five consecutive days) was administered based on clinical considerations.^[31]^ Transfusion is used as a supportive care measure as needed. Plasma exchange, CsA (initial oral dosage of 2.5 mg/kg twice per day), bortezomib (intravenous dosage of 1.3 mg/m^2^ weekly for 1-4 weeks), and MMF (initial oral dosage of 500 mg twice daily) were considered potential alternative options when prior treatment was ineffective.^[1,2]^

### Statistical analysis

Continuous variables in this study are reported as the mean ± standard deviation or median (interquartile range), depending on whether their distribution was normal. For the assessment of between-group differences, the Student's t-test or the Mann-Whitney U test was utilized, depending on the data distribution characteristics. For categorical variables, Pearson's chi-square test and Fisher's exact test were employed to analyze group-level discrepancies. Potential risk factors for AIHA occurrence were identified using univariate Cox analyses, and variables with *P* < 0.1 were further included in multivariate Cox analyses. The cumulative incidence of treatment response and OS was respectively estimated using the Kaplan-Meier method with group comparisons conducted *via* the log-rank test. Cox regression analyses were also used to identify potential predictors of relapse and mortality. All of the tests were two-tailed, and *P* < 0.05 was considered statistically significant. All the statistical analyses were conducted with SPSS version 26 software (IBM Inc, Armonk, NY, USA) and R software version 4.2.2 (Murray Hill, New Jersey).

## Results

### Patient characteristics and incidence of AIHA

A total of 8445 patients underwent allo-HSCT at the Peking University Institute of Hematology between 2013 and 2024, among whom 61 patients with post-allo-HSCT AIHA were retrospectively identified. The clinical data and follow-up records of enrolled patients were complete. The incidence of AIHA following allo-HSCT at Peking University People's Hospital from 2013 to 2024 was 0.72% (6.02 per 10,000 person-years). No statistically significant difference in OS was detected between the AIHA group and the control group (23.0% *vs*. 14.8%, *P* = 0.138). Significant differences in unrelated donors, history of acute GVHD (aGVHD), cytomegalovirus (CMV) infection after allo-HSCT, acute kidney injury (AKI) before AIHA onset, and history of chronic GVHD were observed between groups. Table 1 summarizes the baseline and clinical characteristics compared between the AIHA group and the control group.

**Table 1:** Baseline and clinical characteristics compared between AIHA group and control group

Variables	Control (n = 183)	AIHA (n = 61)	P
Gender, *n* (%)			0.81
Male	129 (70.49)	42 (68.85)	
Female	54 (29.51)	19 (31.15)	
Age (years) , M (Q_1_, Q_3_)	24.00 (8.00, 36.50)	23.00 (8.00, 37.00)	0.86
Follow-up duration (day), M (Q_1_, Q_3_)	915.50 (373.75, 1360.50)	649.00 (222.00, 1257.00)	0.08
MNC (× 108/kg), M (Q_1_, Q_3_)	8.99 (7.42, 10.38)	8.92 (7.38, 10.03)	0.24
CD34 positive (× 10^6^/kg), M (Q_1_, Q_3_)	2.71 (1.77, 4.06)	2.48 (1.60, 3.55)	0.50
Mortality, *n* (%)	27 (14.75)	14 (22.95)	0.14
Underlying disease, *n* (%)			0.40
AML	75 (40.98)	18 (29.51)	
ALL	63 (34.43)	25 (40.98)	
MDS	15 (8.20)	8 (13.11)	
Lymphoma	9 (4.92)	1 (1.64)	
AA	13 (7.10)	5 (8.20)	
Others	8 (4.37)	4 (6.56)	
Prior HSCT, *n* (%)	7 (3.83)	4 (6.56)	0.59
Related Donor, *n* (%)	176 (96.18)	53 (86.89)	0.02
Gender match, *n* (%)			0.65
Match	75 (40.98)	27 (44.26)	
Mismatch	108 (59.02)	34 (55.74)	
HLA match, *n* (%)			0.47
Match	157 (85.79)	50 (81.97)	
Mismatch	26 (14.21)	11 (18.03)	
ABO match type, *n* (%)			0.38
Match	99 (54.10)	31 (50.82)	
Major Mismatch	32 (17.49)	14 (22.95)	
Minor Mismatch	42 (22.95)	10 (16.39)	
Bidirectional Mismatch	10 (5.46)	6 (9.84)	
Source of grafts, *n* (%)			0.21
PB	43 (23.50)	18 (29.51)	
PB+BM	140 (76.50)	43 (70.49)	
Conditioning regimen, *n* (%)			0.68
MAC	170 (92.90)	55 (90.16)	
Non-MAC	13 (7.10)	6 (9.84)	
DLI, *n* (%)	26 (14.21)	6 (9.84)	0.38
aGVHD, *n* (%)	88 (48.09)	12 (19.67)	< 0.001
GVHD level, *n* (%)			
Ⅰ	40 (21.86)	6 (9.84)	
Ⅱ	23 (12.57)	3 (4.92)	
Ⅲ	10 (5.46)	1 (1.64)	
Ⅳ	14 (7.65)	2 (3.28)	
Sepsis, *n* (%)	4 (2.19)	2 (3.28)	1.00
Bacterial infection, *n* (%)	51 (27.87)	22 (36.07)	0.23
Fungal infection, *n* (%)	41 (22.40)	19 (31.15)	0.17
Viral infection, *n* (%)	87 (47.54)	25 (40.98)	0.37
CMV infection, *n* (%)	73 (39.89)	15 (24.59)	0.03
EBV infection, *n* (%)	20 (10.93)	3 (4.92)	0.16
AKI, *n* (%)	4 (2.19)	6 (9.84)	0.03
Liver injury, *n* (%)	20 (10.93)	10 (16.39)	0.26
Hypertension, *n* (%)	12 (6.56)	6 (9.84)	0.57
Diabetes, *n* (%)	11 (6.01)	4 (6.56)	1.00
Heart failure, *n* (%)	5 (2.73)	1 (1.64)	1.00
cGVHD, *n* (%)	23 (12.57)	2 (3.28)	0.04

M: Median; Q_1_: 1st Quartile; Q_3_: 3rd Quartile; AIHA: autoimmune hemolytic anemia; MNC: mononuclear cell count at allogeneic hematopoietic stem cell transplantation (allo-HSCT); CD34 positive: count of CD34 positive cells at allo-HSCT; AML: acute myeloid leukemia; ALL: acute lymphoblastic leukemia; MDS: myelodysplastic syndrome; AA: aplastic anemia; HSCT: hematopoietic stem cell transplantation; HLA: human leukocyte antigen; ABO: ABO blood group; PB: peripheral blood; BM: bone marrow; MAC: myeloablative conditioning; DLI: donor lymphocyte infusion; aGVHD: acute graft-versus-host disease; CMV: cytomegalovirus; EBV: Epstein-Barr virus; AKI: acute kidney injury; cGVHD: chronic graft-versus-host disease.

### Clinical characteristics of patients with AIHA

The baseline characteristics and clinical features of the included patients with post-allo-HSCT AIHA are presented in Supplementary Table S1. Among the 61 AIHA patients, 42 (68.9%) were males, and 19 (31.1%) were females.

Fifty-eight (95.1%) patients were identified as having wAIHA, while the remaining 3 (4.9%) patients had CAS. No patient was identified as having PCH. The median age at allo-HSCT was 23 years (interquartile range [IQR]: 8-37 years), and the median time of AIHA onset was 195 days (IQR: 105-375 days) after allo-HSCT. Fourteen (23.0%) AIHA patients died during the follow-up period, whereas six (9.8%) experienced AIHA relapse. Severe infection-related complications accounted for 12 deaths (85.7%), with the remaining two deaths attributed to hepatic failure and severe hemolysis, respectively. We also conducted the first comparison of available bone marrow morphological examination results among AIHA patients within one year of AIHA onset, and no significant differences were observed.

### Risk factors of AIHA

**Figure 2:**
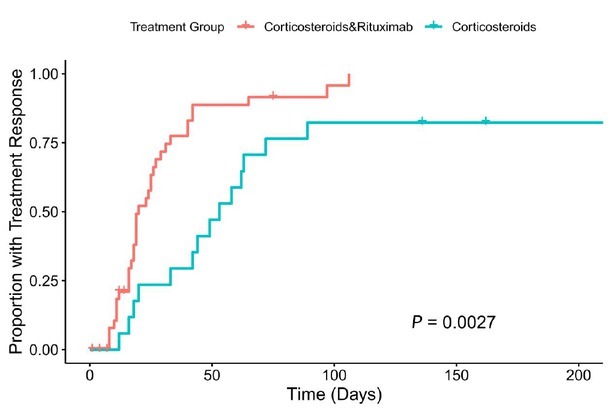
Forest plot of multivariable Cox regression analysis for risk factors of AIHA after allo-HSCT. AIHA: autoimmune hemolytic anemia; allo-HSCT: allogeneic hematopoietic stem cell transplantation; aGVHD: acute graft versus host disease.

Univariate Cox regression identified an unrelated donor as a risk factor for AIHA following allo-HSCT, whereas a history of aGVHD was associated with a reduced risk. Multivariate analysis further revealed that an unrelated donor (HR 2.323, *P* = 0.027) was an independent risk factor for AIHA following allo-HSCT, whereas a history of aGVHD (HR 0.340, *P* = 0.001) was a protective factor. Table 2 shows the results of the Cox regression analyses and Figure 2 presents a forest plot of the multivariate Cox regression results. Analysis of other variables, including sex, underlying disease, conditioning regimens, ABO match type, and others, showed no significant association with AIHA occurrence. Furthermore, in the review of direct triggers for AIHA following allo-HSCT in this study, no AIHA episode was attributed to drugs or autoimmune diseases. Among the 61 AIHA patients, 54 (54/61, 88%) exhibited a clear temporal association with an infectious episode. The infectious etiologies identified included parvovirus B19 (*n* = 2), Mycoplasma pneumoniae (*n* = 3), CMV (*n* = 3), Epstein-Barr virus (EBV, *n* = 1), other bacterial infections (*n* = 5), fungal infections (*n* = 3), and polymicrobial infections (*n* = 37). One patient with CAS was strongly suspected to have developed an AIHA episode in the context of underlying lymphoma relapse.

**Table 2:** Risk factors of AIHA following allo-HSCT

Variables	Univariate			Multivariate		
	HR	95% CI	P	HR	95% CI	P
Unrelated donor	2.668	1.268-5.616	0.010	2.323	1.101-4.902	0.027
aGVHD	0.264	0.132-0.530	< 0.001	0.340	0.180-0.640	0.001

HR: Hazard Ratio; CI: Confidence Interval; AIHA: autoimmune hemolytic anemia; allo-HSCT: allogeneic hematopoietic stem cell transplantation; aGVHD: acute graft versus host disease.

### Treatment and outcomes

**Figure 3:**
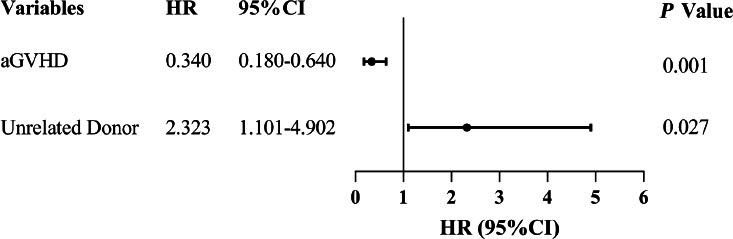
Survival curve for treatment response over time in warm antibody-mediated AIHA patients receiving corticosteroids combined with rituximab and corticosteroids alone. AIHA: autoimmune hemolytic anemia.

Fifty-two (85.2%) patients achieved a treatment response during the treatment course. The median time from onset of AIHA to achievement of CR/ R was 21.5 days (IQR: 16-42 days). All patients with wAIHA received corticosteroid therapy, while all patients with CAD received the standard regimen for RTX. Prednisolone/prednisone in combination with RTX—serving as one of the first-line therapeutic regimens—was shown to increase the probability of achieving CR/ R in wAIHA patients (log-rank *P* = 0.0027, Figure 3), however, this treatment protocol did not improve overall survival (log-rank *P* = 0.422). Among the 18 patients with wAIHA treated with prednisone/prednisolone plus RTX, 14 received the standard RTX regimen, three received the fixed high-dose RTX regimen, and one received the low-dose RTX regimen. Sensitivity analysis for RTX regimens revealed no significant differences in efficacy between the groups (Supplementary Figure S1). With respect to the three patients with post-allo-HSCT CAS, RTX-based treatment achieved treatment response in all cases. Furthermore, other currently recommended therapeutic interventions, including corticosteroids alone and intravenous immunoglobulin administration demonstrated no significant positive effects on treatment response or long-term survival. Two patients who underwent plasma exchange ultimately died, whereas those treated with bortezomib (*n* = 1), CsA (*n* = 1), or MMF (*n* = 2) all achieved a treatment response and long-term survival. However, given the limited number of patients receiving these treatments, the efficacy of these therapeutic options warrants further evaluation. Table 3 presents the major types of therapies and the frequency of patients receiving these therapies during their hospital stay or follow-up period within eight weeks. Moreover, patients who received different treatments did not significantly differ in terms of relapse. Additionally, no significant treatment-related adverse reactions or treatment-related mortality were observed.

**Table 3:** Major treatments used at diagnosis and afterwards in AIHA patients within 8 weeks

Treatments	Week 1	Week 2	Week 3	Week 4	Week 5	Week 6	Week 7	Week 8
Corticosteroid	60 (98.4%)	58 (95.1%)	56 (91.8%)	56 (90.2%)	54 (88.5%)	54 (88.5%)	54 (88.5%)	54 (88.5%)
Rituximab	6 (9.8%)	11 (18.0%)	16 (26.3%)	18 (29.5%)	14 (23.0%)	4 (6.6%)	3 (4.9%)	0
Transfusion	38 (62.3%)	34 (55.7%)	23 (37.8%)	19 (31.1%)	13 (21.3%)	8 (13.1%)	7 (11.5%)	6 (9.8%)
IVIG	45 (73.8%)	21 (34.4%)	6 (9.8%)	3 (4.9%)	2 (3.3%)	0	1 (1.6%)	0
R/CR	0	10 (16.4%)	25 (41.0%)	31 (50.8%)	35 (57.4%)	41 (67.2%)	43 (70.5%)	44 (72.1%)
AIHA relapse	-	-	-	-	0	0	0	0
Death	2 (3.3%)	3 (4.9%)	4 (6.6%)	4 (6.6%)	4 (6.6%)	4 (6.6%)	4 (6.6%)	4 (6.6%)

AIHA: autoimmune hemolytic anemia; IVIG: intravenous immunoglobulin; R: response; CR: complete response.

### Prognostic predictors of patients with AIHA

**Figure 4:**
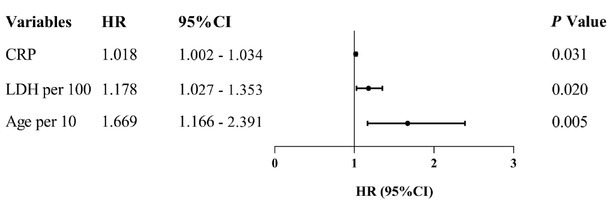
Forest plot of multivariable Cox regression analysis for significant factors associated with mortality in patients with post-allo-HSCT AIHA. allo-HSCT: allogeneic hematopoietic stem cell transplantation; AIHA: autoimmune hemolytic anemia; CRP: C-reactive protein levels at the onset of AIHA; Age per 10: age per 10-year increase; LDH per 100: lactate dehydrogenase levels at the onset of AIHA per 100-unit increased.

According to the results of the prognostic analysis, only the mononuclear cell count at allo-HSCT (HR 1.720, *P* = 0.011) was identified as being associated with AIHA relapse (Supplementary Table S2). Univariate regression analyses screened out the following variables as potential mortality-related risk factors: age, LDH and C-reactive protein (CRP) levels at AIHA onset, and a history of AKI prior to AIHA onset. Multivariate regression analysis indicated that age (per 10-year increase, HR 1.669, *P* = 0.005), LDH levels at the onset of AIHA (per 100-unit increase, HR 1.178, *P* = 0.020), and CRP levels at the onset of AIHA (HR 1.018, *P* = 0.031) were correlated with mortality in patients with post-allo-HSCT AIHA (Table 4, Figure 4).

**Table 4:** Predictors of mortality in patients with AIHA following allo-HSCT

Variables	Univariate			Multivariate		
	HR	95% CI	P	HR	95% CI	P
Age per 10	1.615	1.234-2.113	< 0.001	1.669	1.166-2.391	0.005
LDH per 100	1.162	1.051-1.285	0.003	1.178	1.027-1.353	0.020
CRP	1.020	1.009-1.031	< 0.001	1.018	1.002-1.034	0.031
AKI	6.792	2.110-21.868	0.001			

AIHA: autoimmune hemolytic anemia; allo-HSCT: allogeneic hematopoietic stem cell transplantation; Age per 10: age per 10-year increase; LDH per 100: lactate dehydrogenase levels at the onset of AIHA per 100-unit increased; CRP: C-reactive protein levels at the onset of AIHA; AKI: history of acute kidney injury before AIHA onset.

## Discussion

Despite its rarity, the increased clinical management difficulty and elevated risk of adverse prognoses among post-transplant patients induced by AIHA cannot be overlooked.^[6,15,16]^ The current limited understanding of AIHA after allo-HSCT continues to pose ongoing challenges to its diagnosis and management in clinical practice. With the growing demand for HSCT and allo-HSCT emerging as the primary transplantation modality, detailed investigations into AIHA following allo-HSCT are needed. Herein, we report the incidence, characteristics, risk factors, prognosis, and treatment of AIHA after allo-HSCT based on the largest cohort to date in this field. Furthermore, we systematically identify the prognostic predictors of post-allo-HSCT patients.

The long-term cumulative incidence of AIHA following allo-HSCT in this study was 0.72%, which is lower than that in most previous reports.^[5,6,8,9,10,11]^ This discrepancy might be attributable to differences in underlying diseases, transplant types, transplant-related therapeutic strategies, and the stringent diagnostic criteria for AIHA employed herein. However, this incidence remains significantly higher than that of AIHA in the general population,^[1,32]^ underscoring the necessity for vigilance against post-allo-HSCT AIHA. Regarding the distribution of different AIHA subtypes, we found that AIHA following allo-HSCT remained predominantly wAIHA. These results were not only consistent with findings from relevant post-HSCT AIHA studies,^[33]^ but also aligned with the epidemiological features of AIHA in the general population.^[32]^ The median time of AIHA onset identified in our cohort was 195 days (IQR: 105-375 days) after allo-HSCT, which was generally consistent with the previously reported onset times of AIHA following HSCT.^[34, 35, 36]^ This suggests that AIHA may not present as a short-term complication of HSCT. Instead, monitoring for AIHA in the months to even one year post-transplantation is necessary as part of post-transplant management, particularly in high-risk patients.

Our study revealed a post-allo-HSCT AIHA recurrence rate of 9.8%, and this relapsed outcome appeared to have no evident impact on patients' OS. Given the paucity of relevant reports in the current literature, these findings are of particular interest. The previously reported mortality rates among patients with AIHA after allo-HSCT varied considerably.^[6,11,14,15,16,37]^ Our study found an overall mortality rate of 23.0% in patients with post-allo-HSCT AIHA. Although this percentage was higher than that of the control group (14.8%), the difference did not reach statistical significance (*P* = 0.138). Notably, among the 14 deaths, only one was attributable to severe hemolysis. Severe infection (85.7%) remained the leading cause of death in patients with post-allo-HSCT AIHA. Previous studies have also reported that AIHA may not directly increase post-transplant mortality,^[6,38,39]^ although several studies have suggested otherwise.^[16]^ However, AIHA did increase the complexity of clinical management in this population. Although hemolysis may not be the direct cause of death, most treatments for AIHA—such as corticosteroids and RTX—are immunosuppressive, which significantly complicates infection control in patients with concomitant infections.^[40,41]^ Transfusion requirements in patients with severe AIHA further add to the challenges of clinical management. Moreover, the potential correlation between AIHA and other post-HSCT complications merits careful consideration.^[6,42]^

The complex immune reconstitution process after transplantation makes it difficult to identify clear predisposing factors for AIHA following allo-HSCT. Allo-HSCT significantly affects patients' immune systems and may itself act as a trigger for AIHA.^[1]^ Additionally, multiple post-transplant factors (*e.g*., infections and relapse of underlying diseases) can serve as a "second hit", further exacerbating immune dysregulation in patients and promoting the development of AIHA.^[3]^ By comparing and analyzing the baseline and clinical characteristics between the AIHA and control groups, we found that unrelated donors were an independent risk factor, whereas a history of aGVHD was a protective factor. Unrelated donors in transplant have been reported as a risk factor of post-HSCT AIHA in previous studies.^[3,4,6,13,34]^ Unrelated donors in transplant are associated with delayed immune reconstitution due to lymphodepleting regimens, which may increase the risk of immune dysregulation in transplant recipients.^[43]^ Given that no increased incidence of AIHA was observed in patients who underwent allo-HSCT from donors with HLA or ABO antigen mismatches, directly attributing this phenomenon to the donor's immune system reacting to the recipient's mismatched antigens may lack sufficient justification. Although several previous studies have suggested that aGVHD may be a risk factor for AIHA following allo-HSCT,^[3]^ our study yielded opposite findings. From a pathophysiological perspective, GVHD can damage thymic tissue, resulting in impaired development of self-tolerance,^[44]^ which might increase the risk of AIHA. Notably, the vast majority of patients in our cohort received GVHD prophylaxis and treatment regimens containing MMF and CsA, which are recognized as alternative treatments for AIHA; MMF, in particular, is regarded as a steroid-sparing agent in post-HSCT AIHA, aimed at reducing the duration of concurrent steroid therapy.^[1,3]^ Besides, corticosteroids are among the therapeutic options for aGVHD, which also act as the first-line therapeutic agents for wAIHA.^[45]^ The use of these immunosuppressants may contribute to the lower incidence of AIHA observed in our study and suggests a possibility that aGVHD itself may not directly act as a protective factor for post-allo-HSCT AIHA. Instead, therapeutic interventions for aGVHD might reduce the likelihood of AIHA development. In our study, the incidence of cGVHD was also significantly lower in the AIHA group, which also indicates from another perspective that the prophylaxis/treatment of aGVHD has achieved satisfactory efficacy in patients with AIHA. Furthermore, despite the lack of statistical evidence, given the observed temporal associations between infections, underlying disease recurrence, and the development of AIHA, the impact of "second hits" triggered by these clinical conditions on AIHA remains unclear. Specifically, when such clinical events occur alongside unexplained hemolysis, it is of great necessity to be alert to the potential development of AIHA.

In terms of treatment, the response rate in our study was 85.2%, indicating slightly better efficacy than that reported in previous studies.^[9,14,37]^ We found that corticosteroids combined with RTX—the current first-line recommended therapy for wAIHA—effectively improved the treatment response rate in AIHA patients. RTX functions as an anti-CD20 monoclonal antibody, which achieves selective depletion of B cells through binding to CD20 molecules expressed on the surface of B cells, thereby reducing the production of autoantibodies or allogeneic antibodies. Previous studies have not only confirmed that RTX can significantly increase the remission rate in AIHA patients but also demonstrated its efficacy in treating refractory/ relapsing AIHA.^[46,47]^ Its benefits in patients with AIHA after HSCT have been proved as well.^[7,15,37]^ However, since RTX requires time to deplete B cells, it may not rapidly reduce autoantibody levels. Thus, combination therapy with corticosteroids and RTX is recommended for AIHA patients with rapid disease progression. This regimen could not only rapidly control hemolysis but also suppress antibody production in the long term.^[48,49]^ Although subsequent survival analyses revealed no improvement in OS with this therapy, as noted earlier, AIHA may not directly reduce post-transplant OS. This does not mean that effective therapies for post-allo-HSCT AIHA, including corticosteroids combined with RTX, lack clinical significance. They can ameliorate AIHA conditions, thereby reducing patients' transfusion dependence and shortening hospital stays, which plays a positive role in alleviating the complexity of clinical management and improving quality of life in the post-transplant population. Our results also support the current per spective that bortezomib, CsA, and MMF may also be applicable to patients with post-allo-HSCT AIHA.^[3,9,10,37,50]^ Furthermore, although transfusion and IVIG—two intervention measures that are widely used in clinical practice for treating post-HSCT AIHA, even in critically ill patients—did not yield significant positive outcomes in the present study, they remain potential effective life-saving measures for patients with AIHA following allo-HSCT, considering the favorable patient prognosis observed in this research.^[3,33]^

Few studies have focused on the prognostic factors for AIHA after allo-HSCT, especially the predictors of AIHA relapse. We explored the predictors of AIHA relapse and mortality in patients who underwent allo-HSCT. Mononuclear cell count at allo-HSCT was found to be linked to AIHA relapse. Post-allo-HSCT immune reconstitution is a highly complex process, in which both the quantity and function of mononuclear cells may affect the restoration of immune homeostasis. Mononuclear cells play a crucial role in the immune system: they participate in immune regulation by differentiating into macrophages or dendritic cells and are deeply involved in post-allo-HSCT immune reconstitution.^[51,52]^ A higher mononuclear cell count at allo-HSCT may impact the immune function of transplant recipients by affecting lymphocytes or antibodies, making AIHA patients who achieve remission more prone to recurrence. Previous studies have rarely addressed the field of post-transplant AIHA relapse, and our findings provide new insights for guiding the relapse monitoring and long-term management of post-allo-HSCT AIHA. Multivariate Cox regression further identified age as well as LDH and CRP levels at the onset of AIHA as factors associated with mortality in patients with AIHA following allo-HSCT. Elderly patients may be more severely affected by immunosenescence, which can be associated with a higher risk of post-transplant complications and poor tolerance to treatment. Previous studies have also reported an increased risk of infection-related hospitalization and mortality in elderly AIHA patients.^[53,54]^ LDH is an intracellular enzyme released into the bloodstream when cells are injured or destroyed. LDH levels are generally correlated with the extent of red blood cell destruction and serve as biomarkers for the severity of hemolytic diseases.^[55]^ The association between more severe hemolysis and poor patient prognosis is understandable. Severe infectious complications represent one of the major causes of death in patients after allo-HSCT. In patients with AIHA following allo-HSCT, infections may directly act as a trigger for AIHA and be further exacerbated by the application of immunosuppressive therapy for AIHA.^[3,13]^ This factor significantly increases the difficulty of clinical management and the risk of adverse prognosis for patients. These findings suggest a practical framework for risk stratification. For patients who develop post-allo-HSCT AIHA, particularly elderly patients, those with significantly elevated LDH levels, or those with evidence of infections, clinicians should maintain a high level of vigilance. Such patients may warrant more aggressive upfront therapy, intensive monitoring for infections and organ function, and proactive supportive care to mitigate their high risk of mortality.

This study has several limitations. First, although this cohort of 61 patients with post-allo-HSCT AIHA represented the largest population reported to date, the absolute event numbers, especially the 14 deaths and six relapses, remain small. The impact of the limited sample size and number of endpoint events on our findings should be acknowledged. Consequently, our conclusions require validation in clinical studies based on larger cohorts. Second, the single-center retrospective design inherently limits generalizability. Given the rarity of post-allo-HSCT AIHA, we plan to conduct a collaborative consortium with major domestic allo-HSCT centers to launch a prospective, multicenter study. This initiative will enroll patients treated with diverse graft sources, variable conditioning intensities, and distinct GVHD-prophylaxis platforms, thereby validating our findings. Third, mechanistic studies on post-allo-HSCT AIHA remain limited. Our findings, like those of previous studies, are based on clinical cohort analyses only. Further basic experimental studies are needed to support these clinical observations and deepen understanding of post-allo-HSCT AIHA.

## Conclusion

This study enrolled the largest known cohort of patients with AIHA after allo-HSCT to date, reporting its incidence and clinical features. We revealed that an unrelated donor is a risk factor for AIHA after allo-HSCT, whereas aGVHD and its associated treatments may act as protective factors. We also found that corticosteroids combined with RTX constitutes an effective treatment. Furthermore, we revealed that a higher mononuclear cell count at allo-HSCT is correlated with AIHA relapse, and that older age and higher LDH and CRP levels are associated with mortality in post-allo-HSCT AIHA patients. These findings innovatively highlight key prognostic markers, providing guidance for risk stratification and the management of post-allo-HSCT AIHA.

## Supplementary Material

Supplementary Material Details
