# Supplementary material for: Novel insights from a large cohort: Elucidating incidence, risk factors, treatment, and prognostic predictors in autoimmune hemolytic anemia after allogeneic hematopoietic stem cell transplantation
Source: J Transl Int Med. 2026 Apr 4;14(2):225–36. doi: 10.1515/jtim-2026-0030 (PMC13110459; doi:10.1515/jtim-2026-0030)
Supplement: Supplementary file 1 — Supplementary Material Details [file jtim-2026-0030_sm.pdf]

Supplementary materials

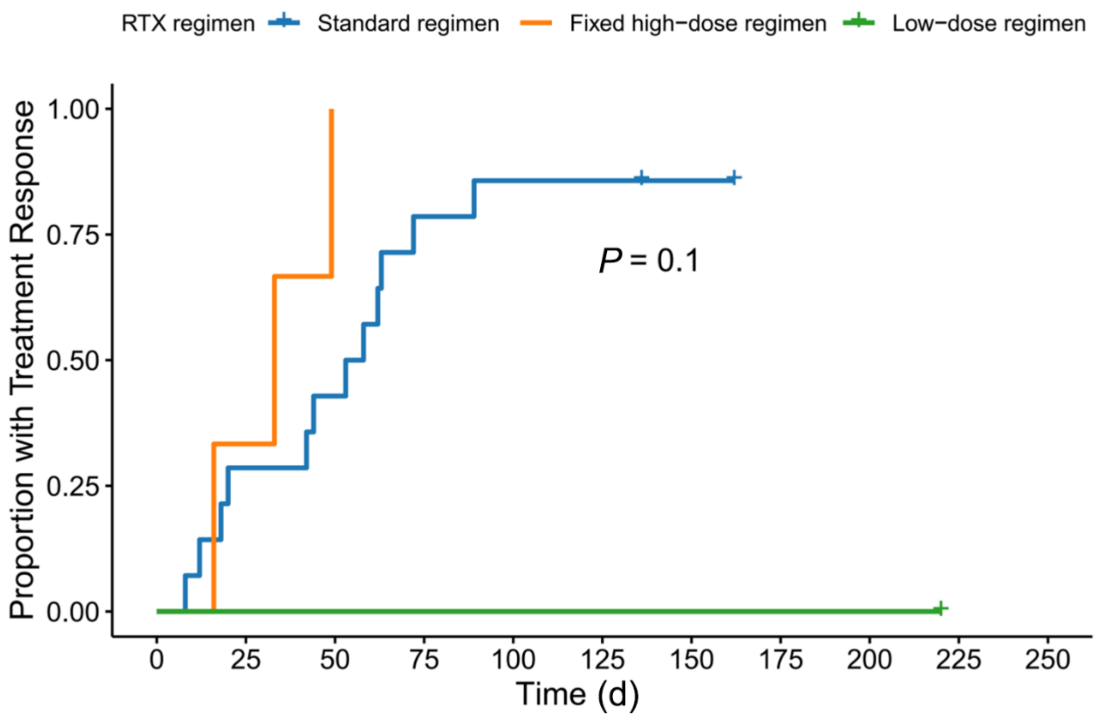

Supplementary Figure S1: Survival curve for wAIHA treatment response over time in patients compared by different rituximab regimens. AIHA: autoimmune hemolytic anemia; wAIHA: warm antibody-mediated AIHA.

Supplementary Table S1: Baseline and clinical characteristics compared between the survival group and mortality group

**Variables**	**Survival (*n* = 47)**	**Death (*n* = 14)**	***P***
Age, M (Q_1, Q_3)	18.00 (6.00, 29.00)	42.00 (29.25, 52.75)	<0.001
Time from HSCT to AIHA onset (day), M (Q_1, Q_3)	208.00 (133.00, 417.00)	133.50 (62.75, 255.75)	0.14
Gender, *n* (%)			0.45
Male	34 (72.34)	8 (57.14)	
Female	13 (27.66)	6 (42.86)	
Relapse, *n* (%)	4 (8.51)	2 (14.29)	0.90
Treatment response, *n* (%)			<0.001
CR	6 (12.77)	0 (0)	
R	39 (82.98)	7 (50.00)	

Variables	Survival (n = 47)	Death (n = 14)	P
NR	2 (4.26)	7 (50.00)	
Follow-up duration (day) , M (Q_1, Q_3)	731.00 (450.00, 1502.00)	155.00 (29.25, 219.75)	<0.001
Underlying disease, n (%)			0.17
AML	14 (29.79)	4 (28.57)	
ALL	20 (42.55)	5 (35.71)	
MDS	3 (6.38)	5 (35.71)	
Lymphoma	1 (2.13)	0 (0)	
AA	5 (10.64)	0 (0)	
Others	4 (8.51)	0 (0)	
Prior HSCT, n (%)	3 (6.38)	1 (7.14)	1.00
Related Donor, n (%)	7 (14.89)	4 (28.57)	0.04
Gender match, n (%)			0.62
Match	20 (42.55)	7 (50.00)	
Mismatch	27 (57.45)	7 (50.00)	
HLA match, n (%)			0.98
Match	38 (80.85)	12 (85.71)	
Mismatch	9 (19.15)	2 (14.29)	
ABO match type, n (%)			0.83
Match	25 (53.19)	6 (42.86)	
Major Mismatch	10 (21.28)	4 (28.57)	
Minor Mismatch	8 (17.02)	2 (14.29)	
Bidirectional Mismatch	4 (8.51)	2 (14.29)	
Source of grafts, n (%)			0.76
PB	12 (25.53)	5 (35.71)	
PB+BM	35 (70.21)	9 (64.29)	
Conditioning regimen, n (%)			0.25
MAC	44 (93.62)	11 (78.57)	
Non-MAC	3 (6.38)	3 (21.43)	
MNC (x 10^8/kg), Mean ± SD	8.79 ± 2.21	8.10 ± 3.33	0.48
CD34positive (x 10^6/kg), M (Q_1, Q_3)	2.52 (1.77, 3.84)	2.31 (1.555, 2.80)	0.37
WBC (x 10^9/L), M (Q_1, Q_3)	4.65 (2.75, 6.68)	3.98 (2.06, 4.75)	0.15
PLT (x 10^9/L), M (Q_1, Q_3)	100 (42.50, 150.50)	33.90 (20.50, 136.00)	0.13

Variables	Survival (n = 47)	Death (n = 14)	P
Hb (g/L), Mean ± SD	57.36 ± 19.53	54.85 ± 20.16	0.68
CRP (mg/L), M (Q_1, Q_3)	6.70 (1.75, 17.75)	17.80 (3.10, 52.60)	0.06
Cre (μmol/L), M (Q_1, Q_3)	46.50 (30.00, 65.50)	80.00 (66.00, 93.00)	<0.001
TBIL (μmol/L), M (Q_1, Q_3)	35.00 (22.65, 49.60)	34.40 (29.30, 67.20)	0.39
DBIL (μmol/L), M (Q_1, Q_3)	10.40 (8.45, 16.65)	15.00 (10.00, 27.80)	0.08
IBIL (μmol/L), M (Q_1, Q_3)	24.40 (13.90, 31.30)	17.45 (11.65, 33.15)	0.55
ALT (U/L), M (Q_1, Q_3)	20.00 (12.00, 32.0)	34.00 (28.00, 94.00)	0.03
AST (U/L), M (Q_1, Q_3)	27.00 (20.00, 35.50)	43.00 (18.00, 73.00)	0.19
LDH (U/L), M (Q_1, Q_3)	368.00 (293.00, 509.50)	452.00 (392.00, 979.00)	0.17
aGVHD, n (%)	7 (14.90)	5 (35.70)	0.23
Bacterial infection, n (%)	16 (34.04)	6 (42.86)	0.55
Fungal infection, n (%)	10 (21.28)	9 (64.29)	0.01
Viral infection, n (%)	16 (34.04)	9 (64.29)	0.04
CMV infection, n (%)	11 (23.40)	4 (28.57)	0.97
EBV infection, n (%)	1 (2.13)	2 (14.29)	0.13
AKI, n (%)	2 (4.26)	4 (28.57)	0.03
Liver injury, n (%)	6 (12.77)	4 (28.57)	0.32
Hypertension, n (%)	3 (6.38)	3 (21.43)	0.25
Diabetes, n (%)	0 (0)	4 (28.57)	< 0.01
Heart failure, n (%)	0 (0)	1 (7.14)	0.23
cGVHD, n (%)	2 (4.26)	0 (0)	1.00

SD: standard deviation; M: Median; Q_1: 1st Quartile; Q_3: 3rd Quartile; AIHA: autoimmune hemolytic anemia; HSCT: hematopoietic stem cell transplantation; CR: complete response; R: response; NR: no response; AML: acute myeloid leukemia; ALL: acute lymphoblastic leukemia; MDS: myelodysplastic syndrome; AA: aplastic anemia; HLA: human leukocyte antigen; ABO: ABO blood group; PB: peripheral blood; BM: bone marrow; MAC: myeloablative conditioning; MNC: mononuclear cell count at allo-HSCT; CD34 positive: count of CD34 positive cells at allo-HSCT; WBC: white blood cell; PLT: platelet; Hb: hemoglobin; CRP: C-reactive protein; Cre: Creatinine; TBIL: total bilirubin; DBIL: direct bilirubin; IBIL: indirect bilirubin; ALT: alanine aminotransferase; AST: aspartate aminotransferase; LDH: lactate dehydrogenase; aGVHD: acute graft-versus-host disease; CMV: cytomegalovirus; EBV: Epstein-Barr virus; AKI: acute kidney injury; cGVHD: chronic graft-versus-host disease.

Supplementary Table S2: Predictors of AIHA relapse after allo-HSCT

Variables	Univariate		
	**HR**	**95% CI**	***P***
MNC	1.720	1.135-2.609	0.011

HR: Hazard Ratio; CI: Confidence Interval; AIHA: autoimmune hemolytic anemia; allo-HSCT: allogeneic hematopoietic stem cell transplantation; MNC: mononuclear cell count at allo-HSCT.
